# Astigmatic Defocus Leads to Short-Term Changes in Human Choroidal Thickness

**DOI:** 10.1167/iovs.61.8.48

**Published:** 2020-07-30

**Authors:** Hosein Hoseini-Yazdi, Stephen J. Vincent, Scott A. Read, Michael J. Collins

**Affiliations:** Contact Lens and Visual Optics Laboratory, School of Optometry and Vision Science, Queensland University of Technology, Brisbane, Queensland, Australia

**Keywords:** astigmatism, choroid, myopia, optical coherence tomography, optical defocus, refractive error

## Abstract

**Purpose:**

To examine the choroidal thickness (ChT) response to short-term with-the-rule (WTR) and against-the-rule (ATR) simple myopic astigmatic defocus, with the response to spherical myopic defocus and clear vision used as control conditions.

**Methods:**

The left eye of 18 healthy adults aged 28 ± 6 years was exposed to clear vision, +3 D spherical myopic defocus, +3 D × 180 WTR, or +3 D × 90 ATR astigmatic defocus for 60 minutes, over four randomly ordered visits, while their right eye was optimally corrected. The macular ChT was measured with optical coherence tomography along the vertical and horizontal meridians before and after 20, 40, and 60 minutes of defocus.

**Results:**

After 60 minutes of defocus, ChT increased by +8 ± 5 µm (*P* < 0.001) with spherical myopic defocus, but varied with simple myopic astigmatic defocus, depending on the axis of astigmatism (*P* < 0.001), increasing by +5 ± 6 µm (*P* = 0.037) with WTR and decreasing by −4 ± 5 µm (*P* = 0.011) with ATR astigmatic defocus. These changes were similar across the vertical and horizontal meridians (*P* = 0.22). The ChT changes were greater than the change during the clear vision control condition (−1 ± 4 µm) for WTR (+5 ± 5 µm, *P* = 0.002) but not ATR (−4 ± 6 µm, *P* = 0.09) astigmatic defocus.

**Conclusions:**

These results provide insights into the human ChT response to short-term astigmatic defocus and highlight a potential difference in the myopiagenic signal associated with the orientation of astigmatic blur.

Astigmatism is a common refractive error in which the retinal image associated with a point source object is formed along two distinct, typically orthogonal, focal planes.^1^ Most infants are born with significant with-the-rule (WTR; horizontal focal plane more myopically focused than the vertical focal plane)^2^ or against-the-rule (ATR; vertical focal plane more myopically focused than the horizontal focal plane)^3^ astigmatism that declines substantially over the first few years of life.^1^ The chronic retinal image degradation caused by infantile astigmatism is thought to hinder the optical cues guiding emmetropization and trigger the development of spherical refractive errors.^4^ This theory is supported by animal models demonstrating altered eye growth with imposed astigmatism,^5^^−^^16^ myopic infants with uncorrected astigmatism exhibiting greater myopia progression during school years compared with their nonastigmatic myopic peers,^17^ and uncorrected astigmatism in preschool children associated with the development and greater progression of myopia during childhood^18^ and early adulthood.^19^ Alternatively, the orientation-dependent retinal image blur associated with astigmatism may aid the visually-guided processes of emmetropization through cues to the sign of defocus.^20^^,^^21^ This theory is supported by studies demonstrating a role for the axis of astigmatism in the development of refractive errors,^4^^,^^22^^−^^24^ with childhood WTR astigmatism associated with less progression of myopia^22^ and ATR astigmatism associated with subsequent development^23^ or greater levels of myopia later in childhood.^4^

Local retinal and choroidal mechanisms have been shown to contribute to the regulation of eye growth, with a thinning or thickening of the choroid typically associated with the mechanisms leading to the development of myopia or hyperopia, respectively.^25^^,^^26^ This study explored the short-term effects of imposed WTR and ATR simple myopic astigmatism on the macular ChT of healthy young adults and compared these changes with ChT responses to spherical myopic defocus and clear vision.

## Methods

Eighteen healthy adults (55% males), with a mean age of 28 ± 6 (range 19–41) years were recruited who were nonsmokers, with normal systemic and ocular health, and were not using any medications. Only participants with identifiable anterior and posterior choroidal boundaries and best corrected visual acuity of 0.00 logMAR or better and anisometropia or hyperopia of less than 1 D were included. The mean spherical equivalent refractive error was −1.9 ± 2.2 D (range −7.00 to +0.75 D), ocular astigmatism was −0.5 ± 0.5 D (range 0.00 to −2.00 D), and axial length was 24.73 ± 1.01 mm (range 22.81 to 26.54 mm) in the left eye. Seven subjects were emmetropes (defined as spherical equivalent refractive error between −0.25 D and +0.75 D, mean spherical equivalent: +0.14 ± 0.29 D, mean axial length: 24.02 ± 0.74 mm), and 11 subjects were myopes (defined as spherical equivalent refractive error ≤−0.50 D, mean spherical equivalent −3.19 ± 1.95 D, mean axial length 25.18 ± 0.91 mm) with no history of any myopia control treatment and wore their habitual spherocylindrical spectacles on the day of each measurement session. The Queensland University of Technology human research ethics committee approved the study. All participants provided written informed consent and were treated in accordance with the tenets of the Declaration of Helsinki.

**Figure 1.**
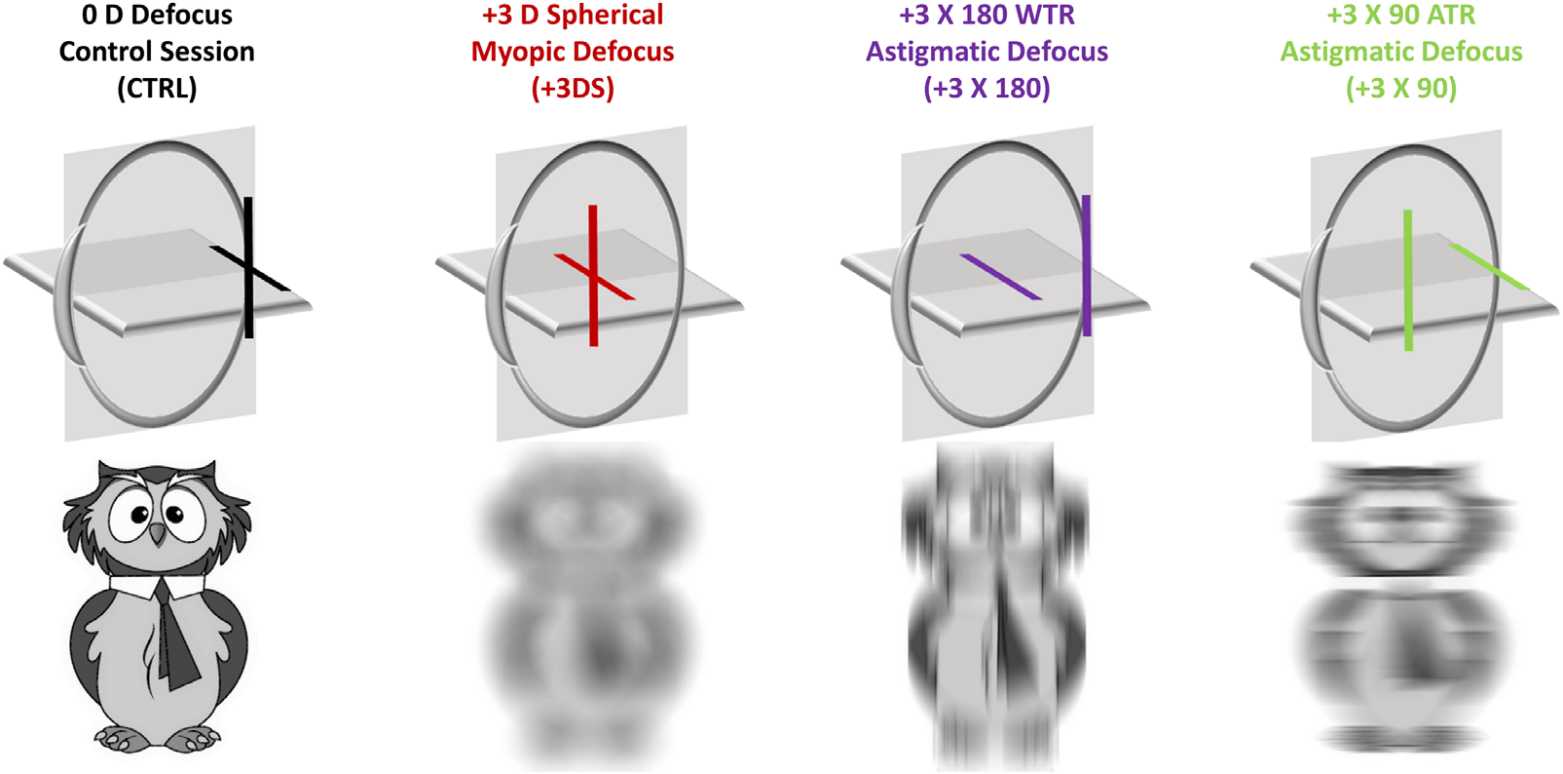
Schematic diagram of the position of vertical and horizontal focal planes relative to the retina across the four experimental sessions (*top row*), and simulation of the effect of each of the defocus conditions over a cartoon image (*bottom row*). The right eye was optimally corrected using sphero-cylindrical trial lenses during each experimental session and was only occluded briefly (∼1–2 minutes) during the OCT imaging of the left eye.

Each participant attended four measurement sessions, each conducted on a separate day in a randomized order, commencing at approximately the same time of day between 9 am to 5 pm, at least two hours after the reported waking time. The left eye's macular ChT was measured before and at 20-minute intervals, during 60 minutes of spectacle-induced +3D spherical myopic defocus, +3D × 180 WTR astigmatic defocus, +3D × 90 ATR astigmatic defocus, or clear vision (Fig. 1) using optical coherence tomography (OCT). Any alcoholic or caffeinated beverages were abstained for four hours before commencement of each session. The room illumination was maintained at 10 lux during each session. Measures of ocular dimensions along the visual axis were assessed with the Lenstar LS 900 (Haag Streit AG, Köniz, Switzerland) optical biometer, and these data were used to rescale the OCT images to account for ocular magnification.^27^^,^^28^

During each experimental session, the participants first performed a 20-minute free-space binocular distant viewing task of watching a movie (projected on a 36° horizontal by 27° vertical screen) at 3 m, with optimal spherocylindrical correction of both eyes (for 3 m distance) in a trial frame. The baseline ChT of the left eye was then measured with OCT. The participants then continued to perform the free-space distant viewing task of watching a movie for a further 60 minutes, while the left eye was exposed to optical defocus (or optimal correction during the control session) and the right eye optimally corrected using spherocylindrical trial lenses, with the ChT of the left eye measured at 20-minute intervals.

**Figure 2.**
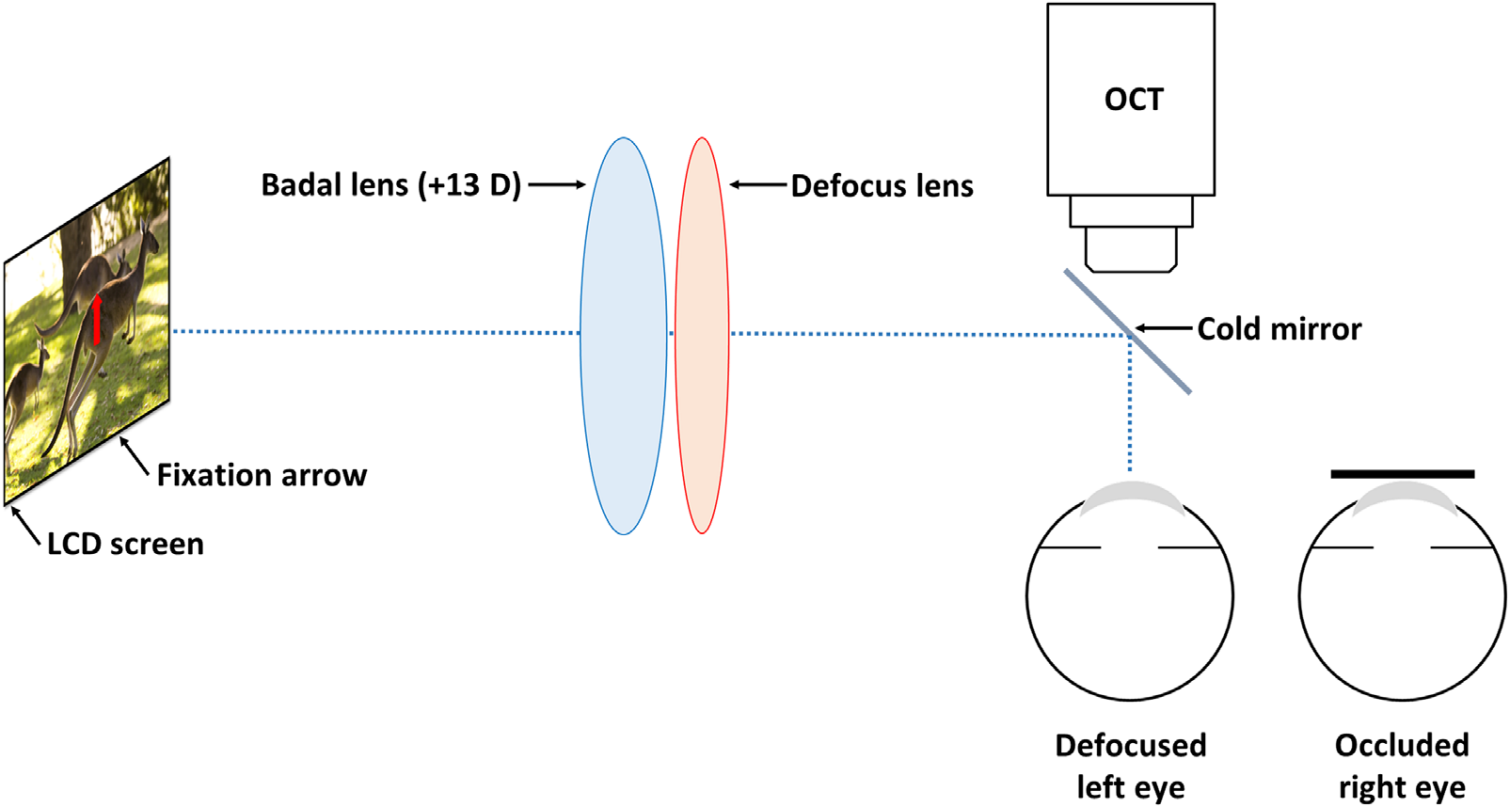
Schematic diagram of the Badal optometer and cold mirror mounted on the OCT instrument for simultaneous exposure of the left eye to defocus stimulus during the OCT imaging of the choroid. The optical axis of the Badal optometer was aligned with the center of the OCT objective lens. The left eye viewed a small arrow (∼0.75°) at the center of a movie displayed on an LCD screen. The fixation arrow was aligned with the optical center of the Badal lens and imaged at optical infinity through the Badal optometer. The defocus spherocylindrical trial lenses were positioned coaxially in front of the Badal lens, with their optical power vertex corrected to the left corneal plane.

Using a Badal optometer and cold mirror mounted on the OCT, the left eye was optimally corrected during all the choroidal imaging performed in the control session and for the baseline imaging performed in the defocus sessions and was exposed to +3D spherical myopic defocus, +3D × 180 WTR astigmatic defocus, or +3D × 90 ATR astigmatic defocus during the follow-up choroidal imaging performed in each defocus session while the right eye was occluded (Fig. 2).

**Figure 3.**
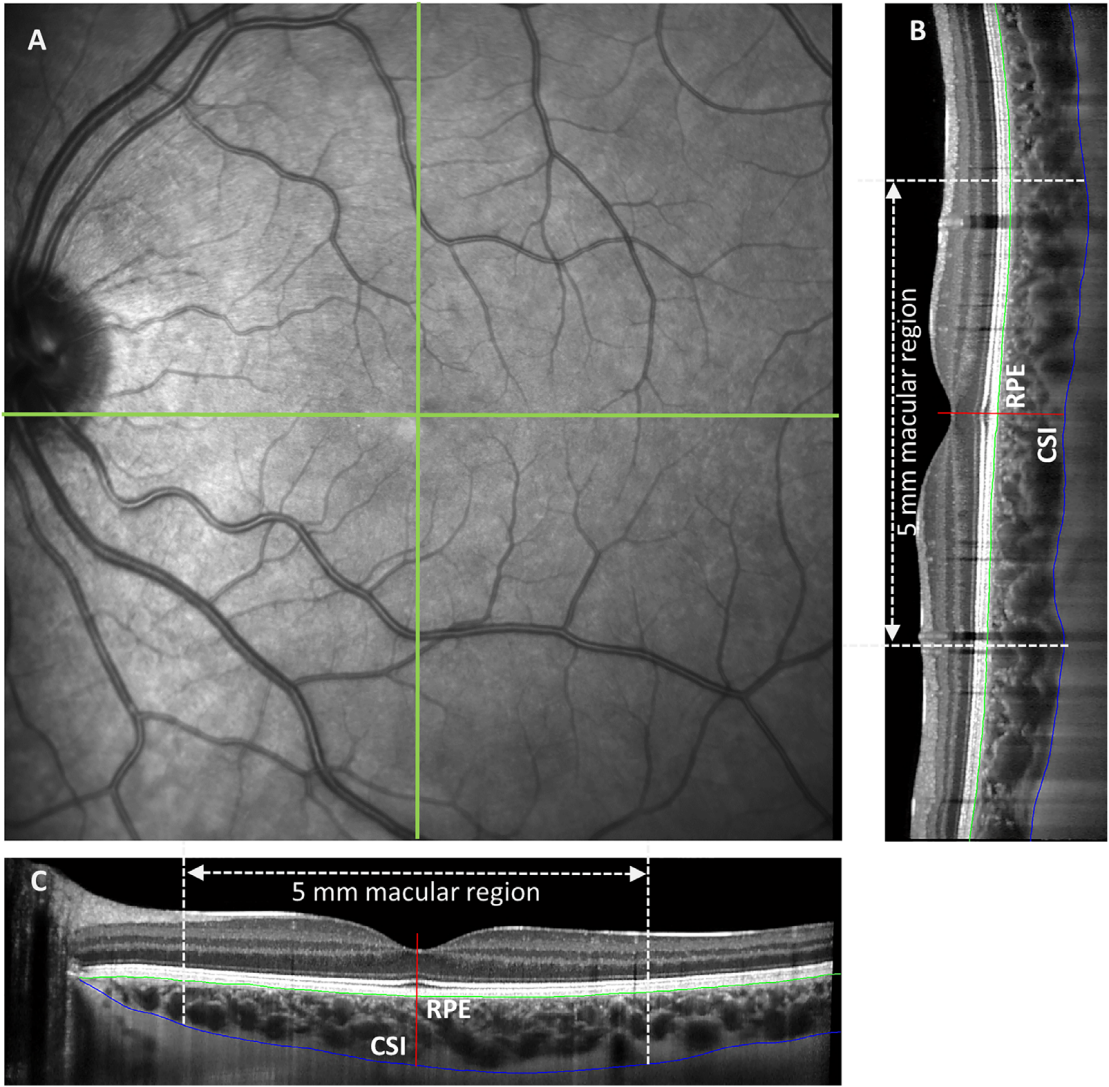
The en-face retinal (**A**) and the vertical (**B**) and horizontal (**C**) B-scan images of a representative subject demonstrating the cross-line enhanced depth imaging OCT scan protocol used in this study (*green lines* in **A**), the position of the fovea at the deepest location of the foveal pit manually marked in the custom-written software (*red line* in **B** and **C**), the anterior (green line in the vertical and horizontal B-scan images) and posterior (*blue line* in the vertical and horizontal B-scan images) boundaries of the choroid, and the central 5-mm macular region across which the thickness of the choroid was averaged. RPE, retinal pigment epithelium; CSI, chorioscleral interface.

Measures of ChT were derived from high-resolution enhanced-depth imaging OCT of the left eye using the Spectralis device (Heidelberg Engineering Co, Heidelberg, Germany). A 30° cross-scan protocol was used and repeated three times at each measurement time point to scan across the vertical and horizontal meridians, centered on the fovea, with each B-scan an average of 100 frames^29^ and exhibiting optimal image quality of minimum 20 dB (Fig. 3). During the OCT imaging, the left eye viewed a small fixation arrow on an LCD screen, presented externally through the Badal optometer and the bright blue internal fixation light of the Spectralis was switched off.^30^ The instrument's follow-up mode allowed all the B-scan images, acquired across all measurement times over the four experimental sessions, to be registered to the first baseline scan of the first visit.

The OCT B-scans collected at each measurement time point were exported and segmented using a semi-automatic procedure.^30^^,^^31^ The anterior boundary of the choroid, defined as the outer border of the hyperreflective line corresponding to the retinal pigment epithelium/Bruch's membrane complex, and the posterior boundary of the choroid, defined as the inner border of the hyper-reflective line corresponding to the chorioscleral junction, were segmented automatically using a custom-written algorithm,^32^ with any errors in segmentation manually corrected by one experienced masked observer. The mean macular ChT, averaged across the central 5-mm region centered on the fovea, was then estimated across the vertical and horizontal meridian (Fig. 3) and averaged across the three B-scans collected at each time point to obtain an average estimate of macular ChT across each meridian before and after 20, 40, and 60 minutes of exposure to defocus.

Repeated measures analysis of variance (ANOVA) was conducted with three within-subject factors of defocus session, time, and meridian and one between-subject factor of refractive error group. When the assumption of sphericity was violated, the Greenhouse-Geisser correction was applied to the degrees of freedom. Bonferroni-adjusted pairwise comparisons were conducted for statistically significant main effects and interactions. Pearson correlation analysis was used to examine the association between age and the mean macular ChT, averaged across the vertical and horizontal meridians, at the baseline measurement of each experimental session, with the changes in the ChT after 60 minutes of exposure to defocus. The within-session coefficient of repeatability (CR, and its 95% confidence interval) for the mean macular ChT, averaged across the vertical and horizontal meridians, was also determined for each defocus session using the method of Bland and Altman.^33^ For this purpose, a one-way ANOVA was carried out using the three repeated baseline measurements of choroidal thickness as the dependent variable and the subject as the independent variable, allowing the within session variability (Sw) and coefficient of repeatability (2.77 × Sw) to be determined for each defocus session. Given that the three baseline B-scans were acquired from an identical choroidal region over a brief period of time (∼1–2 minutes), the observer-related variability in measures of choroidal thickness would be the primary factor contributing to this within session CR.

## Results

The within session CR for the three repeated baseline measurements of macular ChT ranged from 5 to 7 µm across the four defocus sessions (Table), suggesting excellent repeatability for the semi-automatic measurements of macular ChT (and minimal observer-related variability), consistent with previous reports of in vivo macular ChT measurements using these methods.^29^^,^^31^

**Table.** The Mean Macular ChT (µm), Averaged Across the Vertical and Horizontal Meridians, and the Within Session Coefficient of Repeatability (CR, µm) for the Three Baseline Measurements of ChT Estimated Separately for Each Defocus Session (i.e., Separate Days)

Defocus Session	Mean Macular ChT (SD), µm	CR (95% CI), µm
**Clear vision control**	289 (85)	6 (6-7)
**+3 D spherical myopic defocus**	293 (86)	6 (6-7)
**+3 D × 180 WTR astigmatic defocus**	289 (86)	7 (6-7)
**+3 D × 90 ATR astigmatic defocus**	290 (86)	6 (5-6)

**Figure 4.**
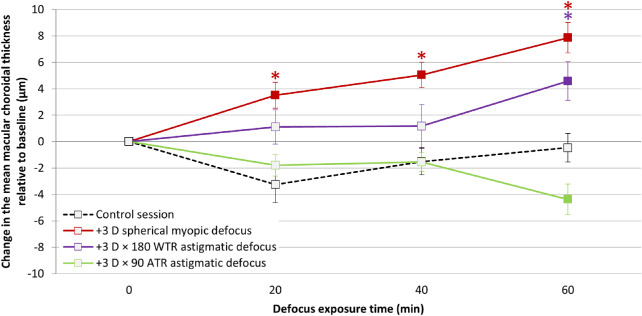
Change in the mean macular ChT of the left eye, averaged across the vertical and horizontal meridians, in response to left eye defocus. Positive and negative values denote thickening and thinning of the choroid, respectively. The filled symbols indicate a significant defocus induced change in the ChT at the corresponding time point compared with the baseline thickness (*P* < 0.05). The *asterisks* indicate a significant change in the mean macular ChT in response to defocus compared with the ChT change observed in the control session at the corresponding time point (Bonferroni adjusted *P* < 0.05). *Error bars* are the standard error of mean.

A significant defocus session by time interaction was observed for measurements of the mean macular ChT, suggesting that the defocus-induced changes averaged across the vertical and horizontal meridians, varied between the four defocus sessions (F_9, 144_ = 9.827, *P* < 0.001), with no significant difference between emmetropes and myopes (defocus session by time by refractive error interaction, F_9, 144_ = 1.568, *P* = 0.13) (Fig. 4).

During the control session, the mean macular ChT, averaged across the vertical and horizontal meridians, did not change significantly over time (−1 ± 4 µm after 60 minutes compared with baseline, *P* = 0.99). However, a significant thickening of the choroid was observed after 20 minutes of exposure to spherical myopic defocus (+4 ± 4 µm, *P* = 0.016), with a further thickening observed after 40 (+5 ± 4 µm, *P* = 0.001) and 60 minutes (+8 ± 5 µm, *P* < 0.001) compared with the baseline ChT.

During the WTR astigmatic defocus session, the mean macular ChT increased gradually (+1 ± 5 µm, and +1 ± 7 after 20 and 40 minutes, both *P* = 0.99), reaching statistical significance after 60 minutes (+5 ± 6 µm, *P* = 0.037). Conversely, the mean macular ChT decreased gradually during the ATR astigmatic defocus session (−2 ± 3 µm after 20 and 40 minutes, both *P* > 0.05), reaching statistical significance after 60 minutes (−4 ± 5 µm, *P* = 0.011). The change in the mean macular ChT was greater after 60 minutes of WTR astigmatic defocus compared to ATR astigmatic defocus (difference of 9 ± 7 µm, *P* < 0.001) (Fig. 4).

The choroidal thickening in response to spherical myopic defocus was greater than the change in ChT during the control session after 20 (difference of 7 ± 6 µm, *P* = 0.002), 40 (7 ± 4 µm, *P* < 0.001), and 60 minutes (8 ± 6 µm, *P* < 0.001) of defocus. However, the choroidal thickening in response to WTR astigmatic defocus was significantly greater than the change observed in the control session only after 60 minutes (+5 ± 5 µm greater, *P* = 0.002). The choroidal thinning in response to ATR astigmatic defocus was not significantly different to the change observed in the control session at any measurement time point (−4 ± 6 µm less after 60 minutes of defocus, *P* = 0.09) (Fig. 4). There was no defocus session by meridian by time interaction (F_9, 144_ = 1.34, *P* = 0.22) or defocus session by meridian by time by refractive error interaction (F_9, 144_ = 0.248, *P* = 0.99), suggesting that the changes in the mean macular ChT associated with 60 minutes of exposure to defocus did not vary between meridians in both emmetropes and myopes.

There was no statistically significant correlation between age and the change in choroidal thickness after 60 minutes during the control (*r* = −0.05, *P* = 0.84), spherical myopic defocus (*r* = −0.45, *P* = 0.06), WTR (*r* = −0.03, *P* = 0.91), and ATR (*r* = −0.03, *P* = 0.90) astigmatic defocus sessions. A significant positive correlation between the mean macular ChT at baseline and the magnitude of choroidal thickening was observed after 60 minutes of exposure to spherical myopic defocus (*r* = 0.5, *P* = 0.03). However, there was no association between the baseline mean macular ChT and the change in ChT during the control (*r* = 0.14, *P* = 0.57), WTR (*r* = 0.18, *P* = 0.46), and ATR (*r* = −0.40, *P* = 0.09) astigmatic defocus sessions.

## Discussion

This study provides the first evidence of changes in human ChT with short-term exposure to astigmatic defocus. After 60 minutes of exposure to simple myopic astigmatism, the ChT, averaged across the vertical and horizontal macular region, varied depending on the orientation of imposed astigmatism. The mean macular ChT increased significantly after 60 minutes of WTR astigmatic blur, with this change also significantly different to the change observed in the control session. Conversely, the mean macular ChT decreased after 60 minutes of ATR astigmatic blur, which was not different to the change observed in the control session. Given the association between changes in ChT and ocular growth,^25^^,^^34^^−^^37^ these changes in ChT associated with short-term astigmatic defocus suggest that orientation specific optical cues provided by astigmatic defocus may have an important role in visually-guided mechanisms regulating human eye growth.

Choroidal thickness changes bidirectionally after short-term exposure to imposed spherical defocus, with myopic defocus eliciting a thickening^30^^,^^38^^−^^42^ and hyperopic defocus a thinning of the choroid.^38^^−^^40^^,^^42^^,^^43^ These spherical defocus-mediated changes in ChT occur within 20 minutes in the subfoveal location^39^^,^^40^ and contribute to alterations in the diurnal variations of axial length^41^^,^^43^ and longer term changes in the axial growth of the eye.^37^ The current study provides further evidence regarding the time course of changes in human ChT associated with spherical myopic defocus beyond the subfoveal location. A significant increase in macular ChT was observed after 20 minutes of exposure to spherical myopic defocus with a further thickening observed over 60 minutes of myopic defocus. This relatively rapid increase in ChT in response to spherical myopic defocus is consistent with other short-term defocus-mediated changes reported in humans that are also thought to contribute to vision-dependent processes regulating eye growth, including changes in the bioelectrical activity of the retinal neurons (within ∼1 second)^44^ and neural adaptation (within ∼4 minutes)^45^ to myopic defocus. Together with recent evidence of local changes in human ChT in response to short-term hemifield myopic defocus,^30^ these retinal and choroidal changes in response to myopic defocus imply that local mechanisms within the eye are involved in the regulation of ocular growth in humans.

The increase in macular ChT after 60 minutes of spherical myopic defocus was also moderately correlated with the baseline ChT, with a greater baseline ChT associated with more thickening in response to spherical myopic defocus. Chen et al.^46^ also reported that the increase in ChT associated with three weeks of orthokeratology (known to induce relative peripheral myopic defocus) in children was associated with the baseline ChT, and a thicker choroid at baseline was also associated with slower eye growth over a 15-month follow-up study in children.^36^ This myopic defocus-mediated increase in ChT proportional to the baseline thickness of the choroid suggests that the pre-treatment ChT may influence the rate of eye growth in myopia control strategies that impose myopic retinal defocus.

Irving et al.^5^ first demonstrated that the mechanisms involved in the control of eye growth respond to complex cues in the retinal image associated with astigmatic defocus. They found that ocular growth was altered in chicks exposed to simple hyperopic or myopic astigmatic defocus, with a greater magnitude of myopia or hyperopia developing in the treated compared to the untreated fellow eye, respectively.^5^^−^^7^ Changes in axial eye growth in response to astigmatic defocus have also been reported in chicks^8^^−^^14^ and monkeys.^15^^,^^16^ In the current study, changes in the macular ChT were observed in response to short-term exposure to simple myopic astigmatic defocus, providing further evidence that orientation-dependent cues associated with astigmatism also alter human ChT. These short-term changes in ChT associated with astigmatic defocus are also in agreement with findings from longitudinal studies reporting an association between childhood astigmatism and later development of refractive errors during school years^4^^,^^17^^−^^19^^,^^22^^−^^24^ providing a likely mechanism that involves the choroid, linking childhood astigmatism and the development of longer-term refractive errors.

In contrast to the choroidal thickening observed with 60 minutes of spherical myopic defocus, the increase in ChT after 60 minutes of WTR astigmatic defocus was not associated with the baseline ChT, possibly suggesting a different signaling pathway involved in the processing of astigmatic compared with spherical defocus. This is further supported by a difference in the time course of changes; a significant increase in ChT in response to WTR astigmatic defocus was observed after 60 minutes, whereas choroidal thickening in response to spherical myopic defocus reached a significant level after only 20 minutes. A slower emmetropization of astigmatic compared to spherical refractive errors in children^2^ and slower defocus-mediated eye growth in monkeys treated with cylindrical compared to spherical lenses^15^^,^^16^ also highlight potential differences in the time course of ocular growth changes in response to astigmatic compared with spherical defocus. Longitudinal studies of refractive error development in children also suggest that astigmatism in early childhood may influence ocular growth in the longer term, only when the children reach teenage years.^4^^,^^19^ Collectively, it appears that the mechanisms mediating the effects of defocus on ChT may differ between spherical and astigmatic defocus, with a longer time constant involved in the cascade of signals processing orientation dependent cues associated with astigmatic defocus.

The fact that the observed choroidal thinning after 60 minutes of ATR astigmatic defocus did not reach statistical significance compared with changes in the control session may also highlight a slower time course of the choroidal response to ATR, compared with WTR astigmatic defocus. Future research investigating ChT changes associated with WTR and ATR myopic and hyperopic astigmatic defocus of a longer exposure period (e.g. over the course of a day) will provide further insights into differences in the time course of the choroidal response to orientation-dependent signals associated with astigmatic defocus.

The changes in axial eye growth with astigmatic defocus vary across studies of animals, ranging from a response favoring the circle of least confusion^7^^,^^10^^,^^11^ to the more myopic or hyperopic principal focal planes,^9^^,^^16^ regardless of the orientation of astigmatic defocus. There is also some evidence for orientation dependent modulation of eye growth in animals exposed to astigmatism. Laskowski et al.^8^ observed that simple hyperopic astigmatism caused myopic eye growth in chicks only if ATR (rather than WTR) astigmatism was imposed, and Kee et al.^16^ found that monkeys experiencing ATR astigmatism in one eye developed more myopia than the fellow eye exposed to WTR astigmatism. The findings from this study do not support a response favoring the circle of least confusion or one of the two principal focal planes. Although the circle of least confusion and the interval of Sturm were myopically defocused during exposure to both astigmatic defocus conditions, an increase in ChT (an expected response to myopic defocus) was only observed after 60 minutes of WTR, but not ATR, simple myopic astigmatism. This differential response of the choroid to short-term WTR and ATR astigmatic defocus suggests that the modulatory eye growth signal associated with astigmatism may vary depending on the orientation of astigmatic defocus.

Given that choroidal thickening is associated with slower eye growth,^35^^−^^37^ the increase in ChT after 60 minutes of WTR myopic astigmatism found in the current study may implicate a potential antimyopiagenic role for WTR myopic astigmatism. The lack of an increase in ChT (and a trend for choroidal thinning) observed in response to ATR myopic astigmatism also implicates a potential myopiagenic role for ATR myopic astigmatism. Although further studies are required to elucidate the long-term effects of WTR and ATR myopic and hyperopic astigmatism upon human ChT and ocular growth, these findings are consistent with previous longitudinal studies demonstrating a differential eye growth trajectory for children with infantile WTR and ATR astigmatism.^4^^,^^22^^,^^23^ Gwiazda et al.^24^ also demonstrated that the axis of ocular astigmatism during infancy may influence eye growth, with infants exhibiting WTR hyperopic or ATR myopic astigmatism developing more myopic refractive errors and those exhibiting WTR myopic or ATR hyperopic astigmatism developing more hyperopia during childhood.^24^ Therefore, the visually-guided mechanisms regulating eye growth may be tuned preferentially to retinal image defocus along a particular meridian, with a defocused or clear retinal image along the vertical meridian associated with astigmatic defocus potentially contributing to mechanisms promoting myopia or hyperopia, respectively. Consistent with this hypothesis, and based on evidence of a weak protective effect of relative peripheral hyperopia on progression of myopia in Chinese schoolchildren,^47^ Atchison and Rosen^48^ have also proposed that the visually driven mechanisms of eye growth may be more susceptible to defocus of the tangential than the sagittal focal plane, with vertically oriented blur potentially eliciting a signal for eye growth and myopia.

Although the exact mechanisms underlying the differential changes observed in ChT associated with WTR and ATR myopic astigmatic defocus are not well understood, we hypothesize that an orientation selective signaling pathway in the retina that is preferentially sensitive to optical defocus along the vertical or horizontal meridian may detect orientation dependent changes in retinal light vergence (and/or image quality) associated with astigmatic defocus and act upstream influencing changes in ChT. Howland^21^ also proposed that the retinal circuitry involved in the visually driven processes of eye growth are sensitive to orientational cues provided by the sagittal and tangential focal planes associated with astigmatism, with a balance between the output signal of these cells required for a normal process of emmetropization. A recent study in chicks also provides evidence for local retinal mechanisms contributing to the modulation of eye growth associated with astigmatic defocus.^14^ Although orientation selectivity in the human retina has not been confirmed, the vertical-horizontal asymmetry in the local spacing of foveal cone photoreceptors (with less spacing vertically than horizontally)^49^ may contribute to orientation selectivity occurring early in retinal light sampling.^50^

There is also compelling evidence from mice and macaques demonstrating retinal amacrine^51^ and ganglion cells^52^ exhibiting preferential sensitivity to vertically or horizontally oriented features.^53^ Therefore, the detection of orientation cues associated with astigmatism may occur early in the visual pathway in the retina. Interestingly, these orientation selective inner retinal neurons have also been demonstrated to be sensitive to spatiotemporal increments (ON response) or decrements (OFF response) in retinal luminance,^51^^,^^53^ with the retinal ON/OFF signaling pathway also known to contribute to vision-dependent mechanisms regulating eye growth in mice^54^ and recent evidence showing bidirectional changes in human ChT with ON/OFF stimuli.^55^ Therefore, it seems plausible that the inner retinal neurons involved in detection of sign of spherical defocus^44^^,^^56^ may also process the orientation specific cues associated with astigmatic defocus and initiate a cascade of (potentially local) signals leading to changes in ChT.

Although the observed changes in ChT during exposure to defocus were small in this study, the magnitude of these changes were consistent with other studies reporting small choroidal thickness changes in humans in response to spherical defocus (typically less than 15 µm).^25^ Furthermore, a range of factors known to have short-term effects on choroidal thickness were controlled carefully in this study (including diurnal effects, near accommodative tasks, physical activity, and ambient lighting) and choroidal imaging was performed simultaneously during, rather than after, exposure to defocus. The potential effect of any short-term variation in choroidal thickness confounding the measured defocus-induced change in choroidal thickness was also minimized by averaging the choroidal thickness across the three B-scans repeated at each measurement time point. The statistical analysis performed in this repeated measures study was also robust with 80% power to detect a 4 µm change in choroidal thickness in response to defocus. Any potential variations in choroidal thickness response to defocus associated with retinal meridian and refractive error were also accounted for appropriately in the repeated measures ANOVA, which yielded a highly significant interaction of defocus session by time in measures of macular choroidal thickness. Therefore the small magnitude changes in choroidal thickness observed during each defocus session are likely to signify a true response to the imposed optical defocus conditions.

In conclusion, macular ChT increased after 60 minutes of WTR simple myopic astigmatic defocus, with a slower time course than the choroidal thickening observed after spherical myopic defocus, highlighting potential differences in the mechanisms mediating the effect of spherical and astigmatic defocus on ChT. The choroidal thickening observed in response to WTR myopic astigmatic defocus did not occur during ATR myopic astigmatic defocus, suggesting that orientation selectivity may exist in vision-dependent mechanisms controlling human eye growth. Whether this orientation selectivity in the choroid response to astigmatic defocus is dose dependent and occurs with WTR and ATR hyperopic astigmatic defocus requires further investigation.
